# The Influence of Anthropogenic and Environmental Disturbances on Parameter Estimation of a Dengue Transmission Model

**DOI:** 10.3390/tropicalmed8010005

**Published:** 2022-12-22

**Authors:** Alexandra Catano-Lopez, Daniel Rojas-Diaz, Carlos M. Vélez

**Affiliations:** School of Applied Sciences and Engineering, Universidad EAFIT, Medellín 050022, Colombia

**Keywords:** dengue, mathematical modeling, parameter estimation, uncertainty analysis, sensitivity analysis, sub-contour box, control

## Abstract

Some deterministic models deal with environmental conditions and use parameter estimations to obtain experimental parameters, but they do not consider anthropogenic or environmental disturbances, e.g., chemical control or climatic conditions. Even more, they usually use theoretical or measured in-lab parameters without worrying about uncertainties in initial conditions, parameters, or changes in control inputs. Thus, in this study, we estimate parameters (including chemical control parameters) and confidence contours under uncertainty conditions using data from the municipality of Bello (Colombia) during 2010–2014, which includes two epidemic outbreaks. Our study shows that introducing non-periodic pulse inputs into the mathematical model allows us to: (i) perform parameter estimation by fitting real data of consecutive dengue outbreaks, (ii) highlight the importance of chemical control as a method of vector control, and (iii) reproduce the endemic behavior of dengue. We described a methodology for parameter and sub-contour box estimation under uncertainties and performed reliable simulations showing the behavior of dengue spread in different scenarios.

## 1. Introduction

Over the past 50 years, arboviral infections transmitted by mosquitoes of the *Aedes* genus have emerged as a significant health problem worldwide [1,2] because of climatic change, human migration, and lack of control strategies [3]. Dengue is endemic in over 100 countries and affects 100 million people annually [4,5]. The control actions can be modeled as parameters or inputs, depending on the type of control: biological control, chemical control, mechanical control (clean-up of the mosquito breeding sites), or vaccination [6,7,8,9,10,11,12,13,14,15]. Note that the World Health Organization (WHO) recommends chemical control only in emergencies to suppress an ongoing epidemic or prevent an incipient one [16]. There are different studies that motivate the study of chemical control using mathematical models to test its effectiveness under several epidemiological conditions [17].

Model parameters that were estimated from real data help more to fit a feasible model that follows real conditions than models with measured-in-lab parameters [18]. Previous papers have included seasonal changes in the mosquito mortality rate [7,19,20], pulse-type inputs that describe vaccination [11] or Wolbachia enhancement [15], and mosquitoes removal from the system [13]. However, in dengue application papers, the authors implemented input parameters obtained from the literature instead of estimating them by fitting the model to available epidemiological data [7,11,13,15,19,20]. Thereby, we identified an opportunity to study the estimation of model parameters and chemical control inputs simultaneously with data from consecutive dengue outbreaks addressing uncertainty issues with reliable confidence intervals.

Note that nominal parameters by themselves are not informative enough about the disease dynamic because they do not address the uncertainty in epidemiological data. Hence, it is necessary to calculate a region inside parameters space where every set of values causes the model output to stay numerically close to the real data [21,22], an approximation of this region is a confidence sub-contour box (CSB) [23]. We implemented global sensitivity methods to characterize the model behavior by identifying how variations in the value of its parameters affect its output. Thus, applying these methods, we focused on pulse input estimations that represent the change in the mosquito mortality rate in specific time intervals using real data from the municipality of Bello (Colombia) during the 2010–2014 outbreaks.

This paper is distributed as follows: first, in the Methodology section, we introduce the study case, describe the mathematical model and the methods to perform the CSB, and the uncertainty and sensitivity analyses (UA/SA). Then, in the Results section, we present the estimated parameters and their CSB for different subsets of epidemiological data. Finally, we present a Discussion section and Conclusions.

## 2. Methods

In the following subsections, we describe the methodology used to study the dengue spread and control dynamics. This is composed of (i) description of a mathematical model with parameters and CSB estimations using real data; (ii) implementation of pulse input signals to perform estimations, in which we consider that the parameter values are not the same during a temporal window; (iii) validation of the CSB quality using UA and SA; and (iv) test the control strategies simulating different pulse-type inputs (vaccination and chemical control).

### 2.1. Characteristics of the Study Area

The municipality of Bello (Colombia) is located in the Andean Mountains at 1450 MASL, with an annual mean precipitation and temperature of 1538 mm and 21.7 °C, respectively [24]. Thus, Bello has appropriate conditions for *Aedes aegypti* reproduction and endemic dengue cases with occasional outbreaks in which the four serotype could circulate at the same time [25,26,27]. In addition, the region has suffered from multiple consecutive dengue outbreaks in some years with a high ENSO effect [28,29], e.g., 2010–2013, 2015–2016, and 2019–2020 [30,31,32,33]. Therefore, the health authorities have taken actions against the spread of the disease: (i) release of mosquitoes with Wolbachia in 2015, (ii) cleaning of breeding sites, and (iii) occasional chemical control [34,35].

Because of the high number of dengue cases reported in 2009 and 2014 in Bello, the municipality performed two aerial spraying. They applied this control action during the 29th epidemiological week in 2010 and the 32nd epidemiological week in 2014 in different locations in the city. The insecticide implemented to manage both outbreaks was Malathion, which has a residual effect for up to 12 weeks and only affects the adult stage of mosquitoes [36]. The reported chemical controls in Bello provided us with information about an extrinsic factor that affected mosquito populations and the number of dengue cases in specific time frames. Thus, we modeled the effect of chemical control actions over dengue cases using pulse inputs (see Section 2.3).

### 2.2. Experimental Data

In Colombia, hospitals report weekly the number of confirmed dengue cases to the National Public Health Surveillance System (SIVIGILA by its initials in Spanish). Pre-established protocols are implemented to monitor classic and hemorrhagic cases that help to identify risk factors and perform control strategies [37]. This study used data from the number of infected people during 265 epidemiological weeks in Bello (Colombia), starting in the 48th epidemiological week in 2009 until the 52nd epidemiological week in 2014; this period covers two dengue outbreaks that occurred in the city between 2009–2010 and 2014. By that time, the population of Bello was about 407,000 inhabitants who were distributed among urban and rural zones [38].

### 2.3. Mathematical Model and Control Actions

Different models have been used to describe dengue spread; these methods depend on the scope and detail that the modeler wants to give to the analysis and control [8,9,10]. Using a model for adult and aquatic phases (eggs, larvae, pupae), we can add different actions to control the models, which allows us to check the effect of these control actions on the mosquito population and disease spread.

In the present study, we implemented a 10-order continuous-time and nonlinear model of dengue spread based on the model given in [39,40], with new parameter (α) and a vaccine control input ($u_{v}$). We define the model parameters in Table 1. All model state-space variables and parameters are non-scaled to conserve their magnitude and biological meaning:

(1) $$\begin{array}{ccc} \dot{E} & = & \delta \left(1-\frac{E}{C}\right)M-\left(\gamma_{e}+\mu_{e}+u_{e}\right)E\\ \dot{L} & = & \gamma_{e}E-\left(\gamma_{l}+\mu_{l}+u_{l}\right)L\\ \dot{P} & = & \gamma_{l}L-\left(\gamma_{p}+\mu_{p}+u_{p}\right)P\\ \dot{M_{s}} & = & f\gamma_{p}P-\frac{\beta_{m}H_{i}M_{s}}{H}-\left(\mu_{m}+u_{m}\right)M_{s}\\ \dot{M_{e}} & = & \frac{\beta_{m}H_{i}M_{s}}{H}-\left(\theta_{m}+\alpha \mu_{m}+u_{m}\right)M_{e}\\ \dot{M_{i}} & = & \theta_{m}M_{e}-\left(\alpha \mu_{m}+u_{m}\right)M_{i}\\ \dot{H_{s}} & = & \mu_{h}H-\frac{\beta_{h}M_{i}H_{s}}{M}-\mu_{h}H_{s}-u_{v}H_{s}\\ \dot{H_{e}} & = & \frac{\beta_{h}M_{i}H_{s}}{M}-\left(\theta_{h}+\mu_{h}\right)H_{e}\\ \dot{H_{i}} & = & \theta_{h}H_{e}-\left(\gamma_{h}+\mu_{h}\right)H_{i}\\ \dot{H_{r}} & = & \gamma_{h}H_{i}-\mu_{h}H_{r}+u_{v}H_{s} \end{array}$$

We based the dengue spread model on the following assumptions: (i) $H_{i}$ corresponds to the reported dengue cases; (ii) the total human population *H* is constant ($\dot{H}=\dot{H_{s}}+\dot{H_{e}}+\dot{H_{i}}+\dot{H_{r}}=const$, which is given by the last four equations in the model), i.e., the human birth and death rates are equal and constant (this is valid for studies that occur in periods when the population does not increase considerably), (iii) the total population of mosquitoes *M* is variable ($M=M_{s}+M_{e}+M_{i}$) because of its short lifetime and the population changes that suffer in short periods, (iv) there is a unique serotype behavior, i.e., it represents an average behavior of the dengue propagation, (v) populations are homogeneously mixed, and the mean behavior is represented (parameters are the average for the entire population), (vi) vertical transmission of the dengue virus is not considered, and (vii) the sample time interval is one week.

We separated the mosquito mortality rate into two parts (as shown in [39]): mortality related to natural environmental conditions ($\mu_{m}$) and mortality related to imposed conditions such as chemical control ($u_{m}$). We modeled $u_{m}$ as a pulse-type input (2), in which *n* is the number of pulses, $t_{0_{c_{j}}}$ is the initial time, $\Delta t_{c_{j}}$ is the duration and $A_{c_{j}}$ is the amplitude of *j*-pulse input; its value can be positive or negative, in which a positive value represents a control effect and a negative value corresponds to a increase in mosquito natality due to, for example, favorable environmental conditions:

(2) $$u_{m}=\sum_{j=1}^{n}u_{m_{j}}\;,\;u_{m_{j}}=\left\{\begin{array}{cc} A_{c_{j}}, & t_{0_{c_{j}}}\leq t\leq t_{0_{c_{j}}}+\Delta t_{c_{j}}\\ 0, & otherwise \end{array}\right.$$

In model (1), the parameter α describes the effect of the virus over the mortality rate of $M_{e}$ and $M_{i}$, as shown in several studies [41,42]. The parameter *f*, in $M_{s}$ expression, represents the sex-ratio at adult emergence [43].

### 2.4. Parameter Estimation

The parameter estimation of mathematical models is a process that assigns optimal values to the parameters to fit simulation outputs to experimental outputs [44]. In this work, we estimated the parameters of the model (1) for three cases associated with the number of pulses $u_{mj}$. The pulse scenarios describe the effect on the mortality of the mosquitoes population caused by chemical control or favorable external conditions:

A. We fit one epidemic outbreak over 93 epidemiological weeks, without pulse-type inputs ($u_{m}=0$ in Equation (2)), i.e., we assume that there were no external dynamics that could affect the mosquitoes population (no chemical control or environmental changes);
B. We fit one epidemic outbreak over 93 epidemiological weeks, but with the addition of one pulse-type input, which describes an external change that perturbs the mosquito population through a chemical control ($u_{m}=u_{m_{1}}$ in Equation (2)). Here, we estimated the parameters of the model and the pulse input together;
C. We fit two epidemic outbreaks over 265 epidemiological weeks (covering 93 weeks of previous cases) and four pulse-type inputs: two positive inputs for two chemical control actions ($u_{m_{1}}$, $u_{m_{4}}$) and two negative inputs ($u_{m_{2}}$, $u_{m_{3}}$) for modeling an increase in mosquito mortality due to some favorable environmental conditions. In addition, in this case, we estimate simultaneously the four inputs and the model parameters.

To perform parameter estimation, we applied the following methods and tools in the mathematical model (1) using the Matlab programming environment: (i) We fit the $H_{i}$ output model with the real data (number of reported cases per week); (ii) We used numerical methods to solve the model during parameter estimation, even for model simulations; (iii) We performed the parameter estimation using the Trust-Region-Reflective algorithm and nonlinear least-squares criterion implemented in Matlab [45], in which the parameter and functional tolerance were equal to $1\cdot 10^{-7}$; (iv) We fixed two initial conditions: $H_{r}\left(0\right)=0$ (the model does not consider recovered humans at the beginning of simulation ) and $H_{i}\left(0\right)=8$ (the number of dengue cases reported in the first week of the outbreak); (v) We defined lower and upper bounds in parameter intervals (see column 3 in Table 2) from reported lab values (see column 2 in Table 2) and then expanded them when the parameter tends toward an interval bound in the estimation process; this makes sense since some parameters could have higher or lower values because of wildlife conditions. All the routines and codes are available in this GitHub repository.

The main problem encountered during parameter estimation in models such as (1) is the reliability of the data. We have only one output data set to fit the parameters, which can lead to different sets of parameters with similar cost functions. Because multiple local minima could exist in our search surface, the estimation algorithm could converge into an unwanted stationary point in the mean squared error surface, resulting in parameter values that are biological or physically impossible [22]. Thus, we performed several parameter estimations to obtain information about the search surface and we started from different initial points trying to avoid local minima. We performed a different number of estimations per case because of computational costs: case A (2420 estimations), case B (1400 estimations), and case C (640 estimations). Then, from all performed estimations, we selected the estimations whose cost function was within a range of 5% of the minimum cost function. With this method, it is possible to take out some local minima presented during estimations and identify a certain family of parameters. For case A, we selected almost 30% of the estimations, whereas we selected 10% for case B and 80% for case C. With this filtered data; we calculated a nominal value for each parameter for the three cases using the statistical median because the median, unlike the mean, is a robust measure of centrality, in which atypical data do not affect the median value as much [46].

### 2.5. Confidence Sub-Contour Box

After we obtained the nominal curves for each case, we aimed to compute CSB for those parameters to address the uncertainty due to misreporting of dengue cases in the data to fit. We were looking for those feasible parameter values that cause the model output to encompass the shape of the real data up to some uncertainty threshold. Following [21], a region in the space of parameters exhibiting that property is called a confidence contour. Unfortunately, the confidence contour usually shows a complex shape for optimization problems of nonlinear models, which, in turn, makes its estimation virtually impossible [21]. Thus, we chose a novel method to compute a rectangular region within the confidence contour starting from some nominal values for the model parameters. Such a region is named the Confidence Sub-contour Box because of its relation to the confidence contour. The algorithm to compute it was implemented in a free Matlab toolbox [47].

The method for CSB computation exploits the linkages between the theory of sensitivity and the model behavior to achieve intervals for the parameters that jointly define a region where the model output shows similarity with the data to fit. In addition, the influence of every single parameter on the model output tends to be equally relevant within this region. We refer the reader to [23] for further reading about the method. To assess the similarity between the fitted data and the model output, we chose an uncertainty threshold level of 30% when computing the CSB for the present study. Such a threshold determines the maximum variation allowed for the model values regarding the data values. In this way, we could provide a contour box for parameters that fit the epidemiological data and its trend.

### 2.6. Sensitivity and Uncertainty Analyses

We performed UAs and global SAs to quantify the uncertainty contribution of every parameter to the model output [48]. In our case, uncertainty was linked to the size of the CSB for each parameter (95% in this study). The UA is a Monte Carlo simulation that graphically assesses the spread of uncertainty from parameters and their interactions to model outputs. The SA attempts to determine the contribution of each parameter to the model output uncertainty. For the SA, we chose two variance-based methods from Saltelli et al. [48] and Xiao et al. [49]; these methods were implemented in a Matlab toolbox [47]. The first method (Saltelli) is particular for scalar model outputs; hence, we used it to quantify the contribution of each parameter to the mean squared error (MSE) function output, i.e., the cost function that quantifies the fit of a given temporal response (from the estimated model parameters) to measured data ($H_{i}$). The idea behind this approach was to identify those parameters that mostly determine the output behavior when all the parameters carry the same uncertainty. In this way, we can suggest that those parameters that had the greatest contribution are important parameters for control actions. The second SA method (Xiao) that we used in particular for temporal responses; thus, we implemented it to explore the uncertainty contribution of parameters to the model output from the estimated confidence sub-contour box.

We focused on case C to perform all the SA procedures; furthermore, for the SA methods, we estimated the indices of the contribution of each parameter alone ($S_{i}$) and the contribution of each parameter and its interactions ($ST_{i}$). For our purposes, the $ST_{i}$ indices were more relevant than the $S_{i}$ indices; however, we used the Saltelli estimator for $S_{i}$ as a reliability indicator because, when estimating the $S_{i}$ index in this way, negative values can be obtained. By definition, sensitivity indices must not be negative, i.e., obtaining negative $S_{i}$ values suggests that an SA with a larger sample size is necessary. We identified the minimum sample size *N* that gives an informative SA result through the convergence of $S_{i}$ and $ST_{i}$, applying the Saltelli method over intervals built from 1% uncertainty for the nominal parameters (this percent gives outputs close to the nominal output).

## 3. Results

In this section, we present the results of parameter estimation for cases A (no pulse input), B (one pulse input), and C (four pulse inputs) alongside their CSBs, which are validated by the UA and SA. Then, we performed a more in-depth analysis of case C, carrying out simulations with the disease control scenarios, including vaccination and chemical control.

### 3.1. Parameter Estimations with Zero, One, and Four Pulse-Type Inputs

**Figure 1:**
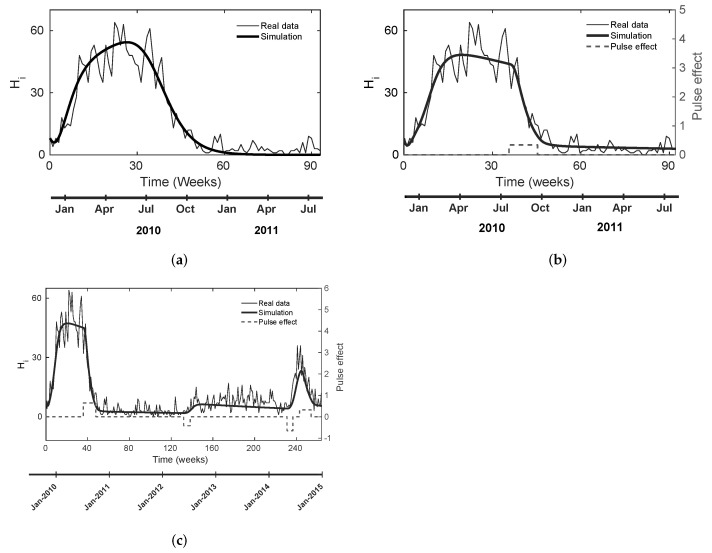
Estimated outputs ($H_{i}$) using the estimated nominal values in Table 2 for cases A, B, and C. The pulse-type input has the same units as the mortality rate (mosquitoes per week). (**a**) Estimated output for one dengue outbreak without pulse-type input; (**b**) estimated output for one dengue outbreak with one pulse-type input; (**c**) estimated output for two dengue outbreaks with four pulse-type inputs.

**Figure 2:**
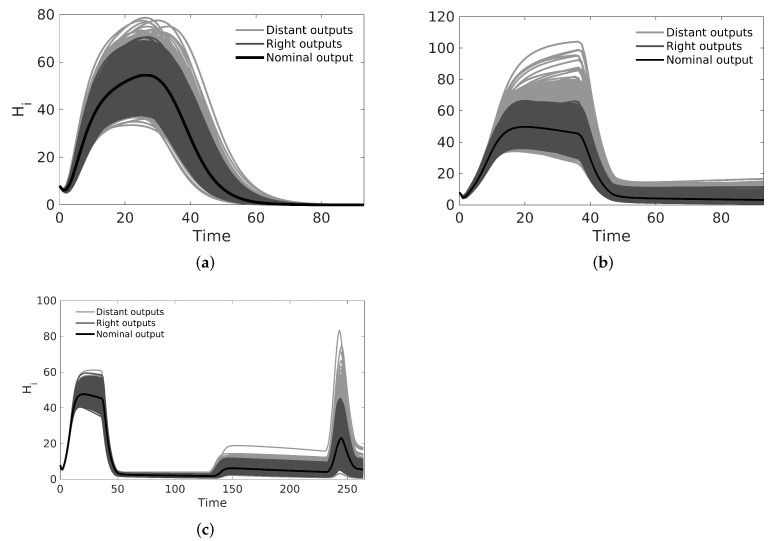
Uncertainty analysis for the dengue model in cases A, B, and C using CSB intervals from Table 2 with 1000 simulations. The CSB method guarantees that at least 95% of curves generated from random combinations of parameters produce outputs classified as ‘right’ outputs. (**a**) Uncertainty analysis for dengue model with non pulses input; (**b**) uncertainty analysis for dengue model with one pulse input; (**c**) uncertainty analysis for a dengue model with four pulse inputs.

As a first approach, we performed multiple parameter estimations using the initial intervals presented in the literature (see Table 2). Then, we obtained the nominal and CSB values for cases A, B, and C, as we stated in Section 2.4 and shown in Table 2. For case A, we identified the following weakness: none of the estimated parameter sets reproduce the endemic behavior, which is clear from disease data (see Figure 1a and Figure 2a), regardless of the number of estimations, i.e., $H_{i}\to 0$ when t→∞.

**Table 2:** Estimated nominal values and confidence sub-contours for parameters and initial conditions in model (1), using zero, one and four pulse-type inputs. The biological intervals reported in the literature [50,51,52] and intervals used in parameter estimation are specified.

			Case A (Zero Pulse Input)		Case B (One Pulse Input)		Case C (Four Pulse Inputs)	
Factor	Biological Interval	Estimation Interval	Nominal Value	CSB	Nominal Value	CSB	Nominal Value	CSB
E(0)	-	(0, 30,000)	17,000	(13,000, 23,000)	21,600	(18,800, 24,900)	9610	(9590, 12,800)
L(0)	-	(0, 30,000)	3400	(2400, 4300)	13,200	(11,000, 19,000)	26,000	(21,000, 35,000)
P(0)	-	(0, 30,000)	5400	(3900, 7500)	12,000	(9700, 16,000)	21,900	(20,900, 22,100)
$M_{s}\left(0\right)$	-	(10,000, 10,000,000)	4,800,000	(4,400,000, 5,600,000)	3,400,000	(2,700,000, 3,800,000)	8,100,000	(7,700,000, 10,000,000)
$M_{e}\left(0\right)$	-	(100, 1200)	1000	(980, 1200)	310	(244, 336)	320	(280, 330)
$M_{i}\left(0\right)$	-	(0, 100)	0.150000	(0.110000, 0.210000)	13	(12, 16)	16	(15, 22)
$H_{s}\left(0\right)$	(0, 400,000)	(0, 450,000)	358,000	(343,000, 414,000)	160,000	(150,000, 190,000)	180,000	(170,000, 200,000)
$H_{e}\left(0\right)$	-	(0, 100)	0.28	(0.21, 0.41)	10	(9, 13)	22	(21, 25)
δ	(65, 165)	(20, 180)	92	(64, 120)	46	(38, 59)	49	(42, 58)
*C*	(6400, 95,000)	(6400, 340,000)	120,000	(95,000, 180,000)	250,000	(238,000, 290,000)	231,000	(199,000, 238,000)
$\gamma_{e}$	(0.6, 2.3)	(0, 2.3)	0.120	(0.099, 0.170)	1.29	(1.10, 1.43)	1.48	(1.28, 1.52)
$\mu_{e}$	-	(0, 1.3)	0.0078	(0.0056, 0.0101)	0.90	(0.84, 1.30)	1.23	(1.14, 1.25)
$\gamma_{l}$	(0.05, 0.5)	(0, 1.6)	0.42	(0.32, 0.59)	0.70	(0.68, 0.88)	0.86	(0.83, 1.07)
$\mu_{l}$	(0.07, 3.22)	(0, 3.22)	2.7	(2.0, 3.9)	1.53	(1.45, 1.88)	1.43	(1.36, 1.67)
$\gamma_{p}$	(0.1, 1)	(0, 1.7)	0.497	(0.415, 0.696)	0.91	(0.75, 0.97)	0.905	(0.902, 1.110)
$\mu_{p}$	(0, 1.4)	(0, 1.4)	1.20	(0.91, 1.75)	0.54	(0.44, 0.65)	0.61	(0.60, 0.66)
*f*	(0.4, 0.6)	(0.3, 0.7)	0.39	(0.29, 0.52)	0.49	(0.42, 0.50)	0.506	(0.449, 0.522)
$\beta_{m}$	(0, 4)	(0, 4)	0.040	(0.032, 0.052)	1.52	(1.50, 1.60)	2.02	(2.01, 2.03)
$\mu_{m}$	(0.06, 0.3)	(0, 0.9)	0.268	(0.244, 0.270)	0.449	(0.449, 0.456)	0.5360	(0.5357, 0.5390)
α	(1, 1.6)	(1, 1.6)	1.03	(1.03, 1.07)	1.44	(1.44, 1.46)	1.4770	(1.4668, 1.4773)
$\theta_{m}$	(0.58, 0.88)	(0.4, 1.0)	0.40	(0.29, 0.51)	0.634	(0.630, 0.660)	0.642	(0.629, 0.643)
$\mu_{h}$	-	(0.00001, 0.0009)	0.000021	(0.000016, 0.000029)	0.000228	(0.000190, 0.000302)	0.000748	(0.000587, 0.000788)
$\beta_{h}$	(0, 4)	(0, 4)	0.227	(0.216, 0.249)	1.43	(1.39, 1.43)	1.43	(1.42, 1.44)
$\theta_{h}$	(0.7, 1.75)	(0.4, 1.8)	0.400	(0.290, 0.430)	0.70	(0.61, 0.72)	0.48	(0.45, 0.49)
$\gamma_{h}$	(0.5, 1.75)	(0.3, 2.0)	0.328	(0.322, 0.381)	1.69	(1.65, 1.69)	1.65	(1.65, 1.67)
$A_{m_{1}}$	-	(0, 2)	-	-	0.48	(0.45, 0.57)	0.69	(0.62, 0.72)
$t_{0_{c_{1}}}$	-	(32, 38)	-	-	35.60	(35.00, 36.00)	35.80	(35.60, 36.80)
$\Delta t_{c_{1}}$	(0, 12)	(0, 12)	-	-	9.7	(7.9, 11.5)	11.99	(11.39, 12.25)
$A_{m_{2}}$	-	(−1.5, 0)	-	-	-	-	−0.46	(−0.54, 0.45)
$t_{0_{c_{2}}}$	-	(120, 134)	-	-	-	-	132	(125, 145)
$\Delta t_{c_{2}}$	-	(0, 12)	-	-	-	-	6	(5, 7)
$A_{m_{3}}$	-	(−1.5, 0)	-	-	-	-	−0.712	(−0.919, −0.709)
$t_{0_{c_{3}}}$	-	(210, 235)	-	-	-	-	231	(227, 233)
$\Delta t_{c_{3}}$	-	(0, 12)	-	-	-	-	5.67	(4.86, 5.86)
$A_{m_{4}}$	-	(0, 2)	-	-	-	-	0.34	(0.32, 0.36)
$t_{0_{c_{4}}}$	-	(240, 260)	-	-	-	-	243	(240, 246)
$\Delta t_{c_{4}}$	(0, 12)	(0, 12)	-	-	-	-	11	(10, 12)

With cases B and C, we obtained parameter values farther from the estimations bounds (see Table 2); these can simulate and reproduce the endemic behavior of the disease (see Figure 1b and Figure 2b). Furthermore, for case C, the model can simulate other real data dynamics, including a new outbreak and chemical control scenarios. For both cases, nominal parameter values of chemical control inputs ($t_{0_{c_{1}}}$, $\Delta t_{c1}$,$t_{0_{c_{4}}}$, $\Delta t_{c4}$ ) coincide with reported values from the Bello municipality and WHO; the values of $t_{0_{c_{1}}}=36$ and $t_{0_{c_{4}}}=243$ in Figure 1c correspond to the 29th epidemiological week in 2010 and the 32nd epidemiological week in 2014, respectively.

Besides the parameters mentioned above, cases B and C show more similar nominal values for parameters α, $\gamma_{e}$, $\mu_{e}$, δ, $\mu_{l}$, $\gamma_{p}$, $\mu_{p}$ or $\gamma_{h}$, whose intervals overlap among them. Note that the value of α is greater than 1 in the CSBs for all three cases. This result suggests the mortality of mosquitoes exposed to the virus ($M_{e}$ and $M_{i}$) to be greater than the mortality of the healthy ones ($M_{s}$).

### 3.2. Estimation of a Sub-Contour Box for Nominal Parameters

**Figure 3:**
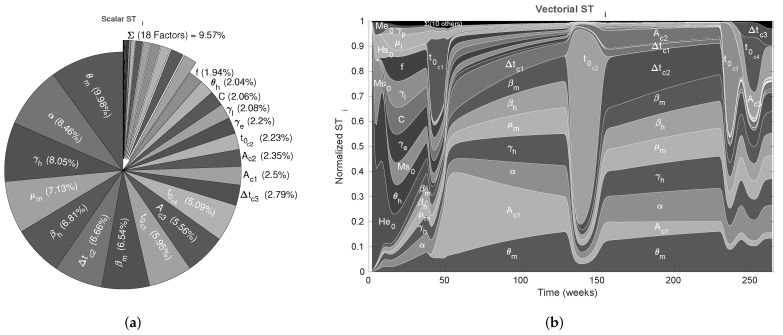
Sensitivity analysis for the CSB intervals. (**a**) The scalar (MSE) SA results for case C and graphical CSB validation using the Xiao method and CSB intervals; (**b**) results of the vectorial SA plotted by time for case C and graphical CSB validation using Xiao’s method with *N* = 6000 [49].

We used the nominal parameters from Table 2 to estimate the CSB intervals for cases A, B, and C (also in Table 2); then, as the first validation for the CSB intervals, we performed a UA for all cases. As expected, following the CSB estimation process, all model outputs showed defined bounds with the same trend of the nominal curve. As shown in Figure 2a, for case A, no curves represented endemic behavior, as in Figure 2b,c (cases B and C, respectively). We also performed an SA validation with the Xiao method for CSB, focusing on case C, which is presented in Figure 3. Note that we obtained a uniform tendency for the pie chart, which constitutes a good sign about successfully estimated CSB intervals, in which most of the parameters have the same relative importance in the respective interval. Furthermore, as shown in Figure 3b, the vectorial sensitivity indices of the parameters ($ST_{i}$ in each time instant of the model output) follow a smooth trend, but it is interrupted in some time intervals, in which the parameters $t_{0_{c_{j}}}$ and $A_{cj}$ reflect the effects of the four pulses.

**Figure 4:**
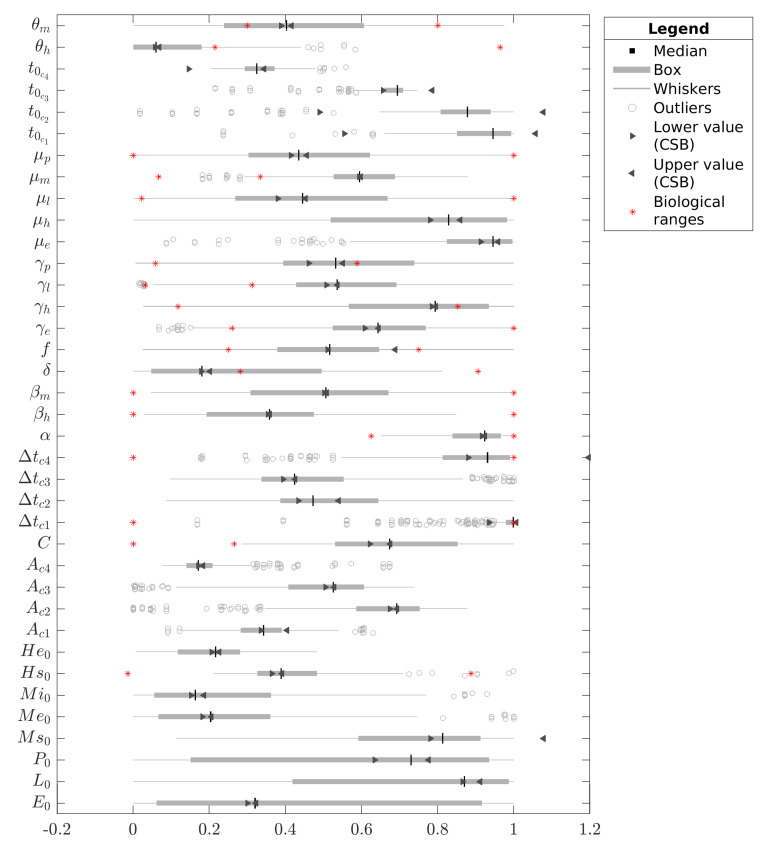
Boxplot for all parameters estimated with the four pulse-type inputs model (Case C). The boxes and whiskers are filtered estimations that define the medians (nominal value), and the red stars represent the biological intervals identified in the literature. All values are normalized according to the minimum and maximum values of the estimation intervals proposed in Table 2.

For the special case of C, Figure 4 compares the CSB intervals with the confidence intervals from the median (see Figure 4). We normalized all data in each case concerning the maximum and minimum bounds of the respective estimation intervals given in Table 2. Note that some CSB intervals are wider than the estimation intervals, e.g., $t_{0_{c_{1}}}$, $t_{0_{c_{2}}}$, $\Delta_{tc1}$, and $M_{s}\left(0\right)$; however, this is not an unexpected result since the CSB interval does not depend on the estimation ranges, and their intervals can exceed the normalization limits [0,1]. Finally, it is remarkable that none of the estimations presented values closer to the inferior bound; indeed, the estimation values were concentrated toward the superior bound.

### 3.3. SA: Parameters That Determine the Model Behavior

After we obtained the nominal values for case C, we performed an SA using a scalar approach to quantify how parameters contribute to MSE fit (estimated output behavior about nominal output). First, we defined the sample size for the SA by performing a convergence of $ST_{i}$ and $S_{i}$ (see Section 2.6 for Saltelli indices). $\sum S_{i}$ and $\sum |S_{i}|$ are almost equal between them so, with a size of *N* = 6000, we can obtain a reliable SA (as stated in the Methods section).

**Figure 5:**
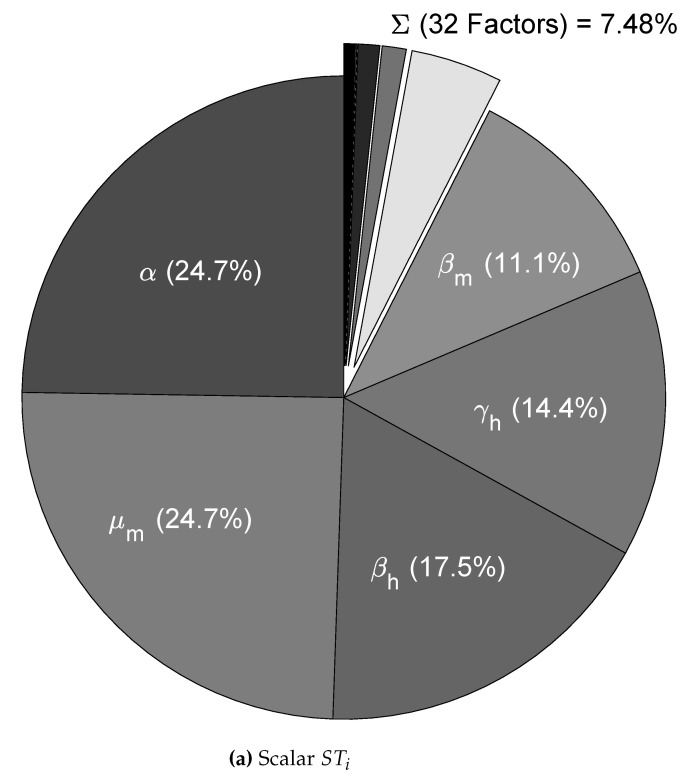

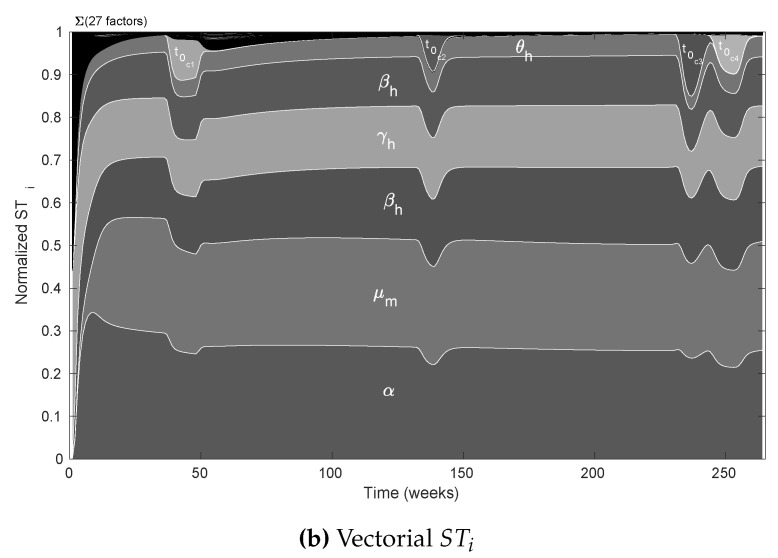
Sensitivity analysis for the dengue model with four pulse-type inputs for case C (with 1% uncertainty in each nominal parameter), using (**a**) scalar and (**b**) vectorial $ST_{i}$ indices from the Saltelli method.

In Figure 5a,b, we summarize the ranking of the most important parameters for the model (1) according to the Saltelli sensitivity method, which quantifies the uncertainty contribution on the scalar MSE output with 1% uncertainty in each parameter (with greater values, the UA gives non-representative outputs). For the scalar indices in Figure 5a, we note that almost 50% of the uncertainty in the output model is caused by the α and $\mu_{m}$ parameters, which are linked to mosquito mortality. Conversely, the transmission rates ($\beta_{h}$ and $\beta_{m}$) and the human recovery rate ($\gamma_{h}$) represent almost 40% of the output variance, and this explains the importance of using mosquito repellents. In addition, note that these parameters take part in the $R_{0}$ expression (see Appendix A), which reinforces the hypothesis that those parameters have the greater potential to change the behavior of the model output, i.e., those are potential targets for control strategies.

The remaining parameters contribute less than 10%, which shows that model behavior is determined primarily by five of the 37 parameters. This finding does not suggest that those less influential parameters are irrelevant to the model; instead, it suggests that we must focus on the highly influential parameters that significantly change the behavior of the nominal curve. For the vectorial indices in Figure 5b, we can see that the importance ranking presented by the scalar indices is almost the same where the parameters related to the pulse-type inputs as $t_{0_{c_{j}}}$ appeared in specific times, affecting the importance of some parameters during the $\Delta t_{cj}$ intervals.

### 3.4. Simulation of Control Strategies

In this section, we simulate three control strategies for case C using nominal parameters estimated for case C, to show some descriptive results from a mathematical model with parameters estimated from real data. These are the considered simulation cases:

- Effect of positive and negative input amplitudes over the vector populations (aquatic and adult stages);
- Variation in pulse-type chemical control input parameters ($A_{c_{j}}$ and $t_{0_{c_{j}}}$, with *j* = {1,4});
- Human immunization (vaccination) as a pulse-type input similar to chemical control (see Section 2.3).

**Figure 6:**
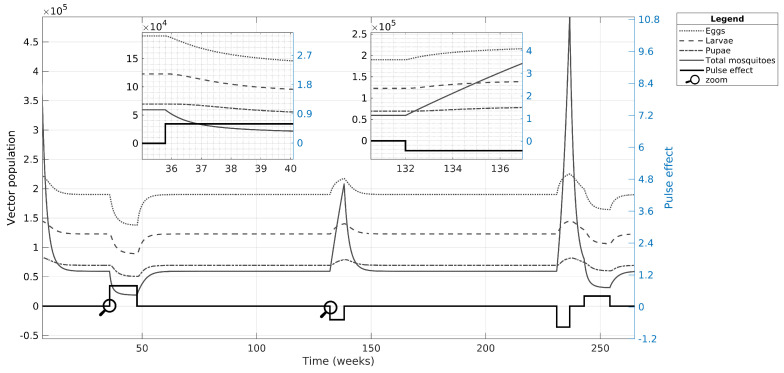
Simulation of case C focusing on the vector population. We used nominal values to observe how the mosquito and aquatic phases are affected by pulse-type inputs. The simulation began in week five because of the quick and significant decrease of the state variables in the vector population. The amplitude of the pulses in the plots is amplified by a factor of 10 for better visualization.

In the first case, we simulated only the nominal value estimated for case C, in which the vector population suffered three major effects, as shown in Figure 6. These effects included one related to the initial conditions and two related to the input effects: (i) the initial conditions of the adult vector population have a pronounced decline (see initial conditions in Table 2); (ii) the chemical control (positive pulse amplitude) reduces all vector populations and, when the residual effect is over, the population gradually returns to their carrying capacity value; (iii) decreasing the mosquito mortality rate (negative pulse amplitude) contributes to increasing the vector population, while the input residual effect remains; and, like the chemical control effect, the population recovers its original value when the input effect is over. Note that the pulse-type control inputs have a large effect on the adult population that spreads to the egg, larvae, and pupae populations with a lower influence.

**Figure 7:**
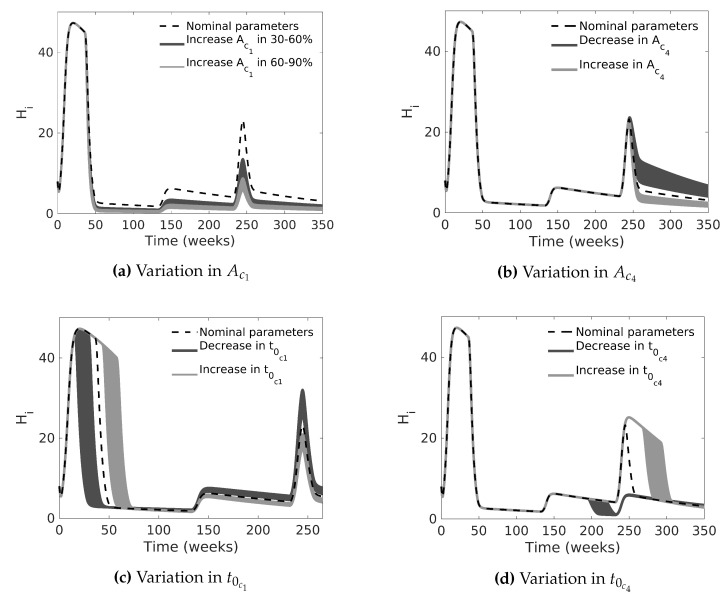
Monte Carlo simulations of the dengue transmission model for the number of infected humans in two scenarios: (**a**) increasing the value of $A_{c_{1}}$ between 30–60% and 60–90% of its nominal value; (**b**) increasing and decreasing the value of $A_{C_{4}}$ between 20% and 60% of its nominal value; (**c**,**d**) late and early chemical control, increasing or decreasing $t_{0_{c_{1}}}$ and $t_{0_{c_{4}}}$ between 20–60% and 10–20% of their nominal values, respectively. For each scenario, we performed 1000 simulations.

In the second case, we fixed all parameters in the nominal values for case C. Then, we performed a Monte Carlo simulation by changing the pulse-type input amplitudes $A_{c_{1}}$ and $A_{c_{4}}$. Figure 7a,b show the behavior of the model by increasing and decreasing the pulse amplitudes between 20% and 60%; note that high amplitude values cause significant mosquito mortality, i.e., they reduce dengue cases, up to a value where they saturate, as expected, whereas low amplitude values are not very effective. In a similar manner, by changing $t_{0_{c_{1}}}$ and $t_{0_{c_{4}}}$, in another Monte Carlo simulation, we can see that the number of infected people decreases when chemical control starts earlier; otherwise, the outbreak increases (see Figure 7c,d).

**Figure 8:**
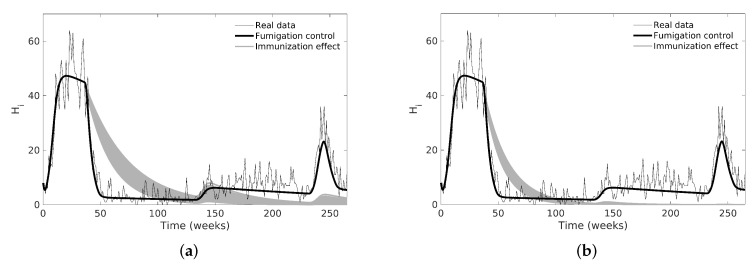
Dengue model simulations using estimated nominal values and immunization control by vaccination. We removed chemical control pulses for immunization simulations and left those that represented the mosquito population increase. (**a**) immunization of the 10 to 20% of total human population; (**b**) immunization of the 20 to 30% of total human population.

Finally, for case C, we evaluated the vaccination action over the number of $H_{s}$, so we eliminated the chemical control inputs and added vaccination control while keeping the mosquito mortality constant. The vaccination is represented by the parameter $u_{v}$ (see Section 2.3), which is implemented as a pulse-type input that moves a certain proportion of people from $H_{s}$ to $H_{i}$ during a period (see Figure 8). We simulated two scenarios with immunization rates between 10–20% and 20–30% per week and per 10 weeks in the same 37th week of chemical control, as shown in Figure 8; we fixed all parameters that were not related to vaccination. Note that the first outbreak is larger in this case than when using chemical control, but the second outbreak is reduced or eliminated.

## 4. Discussion

In the present study, we adapted the mathematical model (1) and estimated its parameters considering scenarios with and without pulse-type inputs. We identified diverse dynamics among the three studied cases: we realized that case A (no pulse-type input) provides some estimated parameters anchored to estimation limit ranges (see Table 2), e.g., α, $\theta_{m}$, $\theta_{h}$, and $\mu_{e}$. Those estimated values suggest that the estimation algorithm still might find a better minimum outside the estimation intervals, i.e., in a region with a lesser biological sense; moreover, the model does not provide endemic behavior, as shown in Figure 1a. Conversely, both cases B and C did not present anchored parameters, as presented in case A (see Table 2), and they did display the endemic response (see Figure 1b,c).

The model behavior in the three scenarios makes sense from a biological perspective because Bello authorities performed fumigation to mitigate the spread of dengue. Thus, if we did not consider a pulse input in the model, the fumigation effect would be included as an implicit component of the estimated value in $\mu_{m}$ and other parameters. We highlight that, in case A ($u_{m}=0$), we could not reach an endemic behavior, regardless of the number of estimations we performed. We hypothesize that the lack in endemic behavior is caused by the real data behavior that the model itself could not reproduce without pulse inputs, where dengue cases increased and decreased rapidly during January 2010–July 2011 outbreak because of the anthropogenic or environmental disturbances. For the case of B ($u_{m}=u_{m_{1}}$), we incorporated the effect of the exogenous action, allowing the model to reproduce an endemic scenario after the first outbreak.

For the case of C, the model had several available pulses ($u_{m}=u_{m_{1}}+u_{m_{2}}+u_{m_{3}}+u_{m_{4}}$). This means that the model can fit the rapid changes in real data caused by anthropogenic and environmental disturbances such as fumigation or climatic variations. Hence, we hypothesize the pulses act as excited signals and theoretically lead to a better estimation [53] that could reproduce trends in epidemiological data overcoming limitations of the non-pulse scenario (case A). Even more, future research could study the changes in the model equilibrium caused by the presence and absence of pulse inputs, which is a theoretical point of view to identify mathematical characteristics that we did not address in this applied study.

After we obtained nominal values for cases A, B, and C, we estimated CSB (see Table 2, columns 5, 7, and 9) as described in Section 2.5. Figure 4 shows the CSB and their relation with the biological and initial estimation intervals, which allows us to identify that some parameters are outside of biological enclosures (the respective CSB are also outside of biological enclosures). We performed both UAs and SAs to assess the relevance of the contours we obtained from the CSB; we can see graphically these analyses in Figure 2. In the first example, for an uncertainty level of 30%, the UA shows that the CSB intervals define a band that includes the nominal curve and real data for each case without non-representative curves. This case implies that CSB intervals are suitable for describing the uncertainty related to several processes, such as misreporting in real data and numerical errors during parameter and interval estimations, among others. Moreover, the bands we obtained for case A, following the same behavior of the nominal curve, never showed the endemic behavior. Conversely, the UA of cases B and C provides both the outbreak and endemic behavior, which shows an effect of pulse-type inputs on both parameter estimation and interval estimation.

As the next step for assessing the CSB method, we performed an SA focusing on case C to quantify how much each parameter contributes to the uncertainty in the model by varying parameter values over CSB intervals. The results are presented in Figure 3a,b, which correspond to the scalar and vectorial SA, respectively. The vectorial analysis is time-dependent, so the $ST_{i}$ indices are calculated over the set of model output for each time step in the simulation; thus, we can determine the relative contribution of the parameters at each time. From vectorial $ST_{i}$, we noticed that the CSB intervals define a region where most of the parameters play an important role, at least from intervals where one could expect this behavior, i.e., the initial conditions are relevant at the beginning of the simulation but irrelevant when the model tends to equilibrium.

Note that mathematical models are not perfect representations of a phenomenon; thus, all parameters are unlikely to achieve high relevance in SA using the CSB approach. However, we should not remove from the model all non-sensitive parameters. Instead, we could fix them at any value for a future work in which we want to identify those dynamics. Using the vectorial SA, we can identify that the pulse-related parameters ($u_{m_{j}}$, $t_{0_{c_{j}}}$ and $\Delta t_{c_{j}}$) are relevant for the time intervals where they are defined, disturbing the ranking of relevance and taking the model out of a stationary tendency (see Figure 2). Additionally, we found that parameters related to aquatic states are relevant only at the start of the outbreak because they do not contribute to the stationary dynamics of the model. We hypothesize that early dynamics of the aquatic states cause a pulse effect over the mosquito population at the beginning of the outbreak. The early pulse effect could generate an increase in the vector population and, therefore, an increase in infections that led to the first outbreak. Thus, for future analyses, we suggest performing estimations with an initial pulse-type input.

The scalar $ST_{i}$ indices that are summarized in Figure 3a were obtained using the Xiao method, whose basis relies on distance components decomposition, i.e., it is a method that allows us to synthesize all the information from the vectorial SA into a single scalar SA index. Hence, Figure 3b constitutes a rapid insight into the relevance of each parameter throughout the entire simulation. The goal of the CSB approach is to achieve intervals such that all the parameters have nearly the same contribution to the model output; however, this was not possible in our study. Thus, readdressing the discussion in the previous paragraph, we attribute the non-uniformity of the SA for CSB to the following causes: (i) the model is an approximation of reality and does not contemplate all spread components and variables; (ii) the CSB is an approximation and only considers a sub-contour box and does not consider the confidence contour itself; and (iii) even with a significant amount of computational time, there are numerical and computational limitations to approximate the CSB.

After performing the SA to validate the CSB intervals, we implemented another SA approach, based on the modified Saltelli method for MSE outputs, to assess parameter influence. This approach allowed us to identify the parameters with the highest potential for changing the nominal output behavior when all the parameters have the same uncertainty level. We chose a unique uncertainty level such that it also defined a band that enclosed the real data. Thus, we determined parameters related to mosquito mortality (α and $\mu_{m}$), infection interactions ($\beta_{h}$ and $\beta_{m}$), and human recovery ($\gamma_{h}$) has the greatest influence in model outputs.We followed these non-classical methods to identify the most important parameters in the model dynamic (UA and SA), which represent, to some extent, a practical identifiability analysis, as proposed by Lizarralde-Bejarano et al. [40]. Therefore, alongside a classical analysis, as presented in the Appendix A, we can reinforce the importance of mortality, recovery, and transition rates in the dengue model behavior. Furthermore, $t_{0_{c_{i}}}$ parameters had a considerable effect on the model output in specific time intervals. It is remarkable that the estimated amplitude for the third pulse input ($A_{m3}$) always led to a negative total mortality rate for the model ($A_{m3}+\mu_{m}$), then it did act as a positive external flow of susceptible, exposed and infected mosquitoes. Regarding such behavior, we hypothesize the rapid increase of dengue cases at the start of the last outbreak to be also related with migration of exposed and infected mosquitoes from neighboring areas, which makes sense, in this case, since Bello is part of a cluster of highly conurbated municipalities. These are significant results since they allow us to propose effective control strategies and suggest that using pulse-type inputs, which change mosquito mortality, could be an appropriate strategy to model extrinsic perturbations.

After performing the UA and SA to validate CSB and estimated nominal parameters, we implemented the pulse-type inputs into the model to determine more information about dengue spread dynamics in three simulation scenarios. In Figure 6, we identify a direct effect of the extrinsic conditions on the mosquito population. Indeed, the pulse-type inputs generate knock-down effects on the vector population, where the negative and positive pulses act as outbreak starters or finishers because of the increase or decrease in the mosquito populations, respectively.

Figure 7a,b show that the amplitude of chemical control input is a determining factor that reduces the outbreak, but the high intensity of insecticide spraying does not produce any effect after a certain value. Figure 7c,d show that late chemical spraying increases the dengue incidence. The simulations provide information about the consequences of performing control in the wrong moment. Figure 7c shows that, if the Health Surveillance Office had applied the chemical control before the first outbreak that occurred in Bello (following the endemic channel criteria proposed by Lizarralde-Bejarano et al. [52]), the dengue outbreak would not have been as serious. We show the same result in Figure 7d for the second outbreak.

In Figure 8, we compared the effect between chemical and vaccination control strategies modeled as pulse inputs, where a vaccination campaign is performed as a long-term strategy in a specific time interval instead of considering it as a constant value over time. As shown in literature, the vaccination is a better option than chemical control in long-term implementation [54] because the disease incidence could disappear from the population if we inoculate enough individuals.

Finally, we implemented pulse-type inputs to estimate and simulate extrinsic conditions, e.g., a decrease in mortality rates because of chemical control. This method could simulate other control strategies such as vaccination, cleaning up breeding sites, Wolbachia introduction into the mosquito population, or biological control. The definition and implementation of these inputs could vary according to the environmental dynamics or by the researcher’s criteria, e.g., instead of a pulse input, we could use a ramp, stairs, or sine curves, among others. Thus, future research could introduce and change these input signals to study the effect of the disease dynamics and the associated uncertainty.

## 5. Conclusions

Dengue spread is related to extrinsic phenomena, including control strategies and environmental conditions. From this premise, we implemented a dengue spread model that considers control strategies (chemical control) and extrinsic phenomena (climatic changes) as pulse-type inputs. This model definition led us to estimate parameter values and CSB. In this process, we determined that the most important parameters in the model are those related to mosquito mortality ($\mu_{m}$ and α), which validates the importance of pulse-type inputs involved in mosquito mortality and vector control strategies. Furthermore, we recommend including pulse-type inputs to estimate parameters and simulate consecutive outbreaks because, with this approach, it is possible to separate anthropogenic or environmental disturbances (chemical control or climatic changes) in specific time windows.

Finally, we identified interesting research questions for a future research, e.g., more research is necessary to determine (i) how to assess the control actions (chemical, mechanical, or biological) in specific time intervals from estimated parameters and their CSB for different geographical regions, (ii) how estimated negative pulse-type control inputs are correlated with environmental conditions, (iii) how to adapt the model to other mosquito-borne diseases (chikungunya or zika), (iv) how to test the impact of the introduction of mosquitoes with Wolbachia bacteria in specific moments on model parameters, or (v) how to evaluate the effect of lower-order models in parameter estimation.

## Figures and Tables

**Table 1:** Definition of state variables, parameters, and input variables for the mathematical model (1). The input that describes mosquitoes growth ($u_{m}$) is defined by three parameters ($A_{c_{j}}$, $t_{0_{c_{j}}}$, and $\Delta t_{c_{j}}$) in Equation (2); in addition, $u_{m}$ could be formed by multiple *j* pulse-type inputs as described in Section 2.4. Note that, for all factors, the time unit is one week, and the rate unit is [week^−1^].

Factors	Description	Factors	Description
*E*	Number of eggs	δ	Oviposition rate
*L*	Number of larvae	*C*	Egg carrying capacity
*P*	Number of pupae	$\gamma_{e}$	Egg to larva transition rate
$M_{s}$	Number of susceptible mosquitoes	$\mu_{e}$	Egg mortality rate
$M_{e}$	Number of exposed mosquitoes	$\gamma_{l}$	Larva to pupa transition rate
$M_{i}$	Number of infected mosquitoes	$\mu_{l}$	Larvae mortality rate
$H_{s}$	Number of susceptible humans	$\gamma_{p}$	Pupae to mosquito transition rate
$H_{e}$	Number of exposed humans	$\mu_{p}$	Pupae mortality rate
$H_{i}$	Number of infected humans	*f*	Fraction of females that emerges
$H_{r}$	Number of recovered humans	$\beta_{m}$	Transmission coefficient human-mosquito
*M*	Total number of mosquitoes	$\mu_{m}$	Mosquito mortality rate
*H*	Total number of humans	α	Change in $\mu_{m}$ due to virus infection
$u_{e}$	Egg control input rate	$\theta_{m}$	Extrinsic incubation rate
$u_{l}$	Larvae control input rate	$\mu_{h}$	Human mortality rate
$u_{p}$	Pupae control input rate	$\beta_{h}$	Transmission coefficient mosquito-human
$u_{m}$	Mosquito control input rate	$\theta_{h}$	Intrinsic incubation rate
$u_{v}$	Vaccine control input rate	$\gamma_{h}$	Human recovery rate
		$A_{c_{j}}$	Mosquito control pulse amplitude
		$t_{0_{c_{j}}}$	Mosquito control initial time
		$\Delta t_{c_{j}}$	Mosquito control pulse width

## Data Availability

Not applicable.

## Appendix A. Reproductive Number R 0 and Equilibrium

In this section, we focused on the $R_{0}$ estimation using the next generation matrix as described in Lizarralde-Bejarano et al. [40], Khan and Fatmawati [55]. For the model (1), we assumed the control inputs as $u_{v}=0,\;u_{e},\;u_{l},\;u_{p},\;u_{m}=0$ because those are modeled as temporal disturbances according to the designed signal (see Equation 2). Then, the disease-free equilibrium is

$$\begin{array}{ccc} \left\{E^{*},\;L^{*},\;P^{*}\right\} & = & \left\{C\left(1-R_{m}\right),\;\frac{\gamma_{e}}{\gamma_{L}+\mu_{L}}E^{*},\;\frac{\gamma_{L}}{\gamma_{p}+\mu_{p}}L^{*}\right\}\\ \left\{M_{s}^{*},\;M_{e}^{*},\;M_{i}^{*}\right\} & = & \left\{\frac{\gamma_{p}}{\mu_{m}}P^{*},\;0,\;0\right\}\\ \left\{H_{s}^{*},\;H_{e}^{*},\;H_{i}^{*},\;H_{r}^{*}\right\} & = & \left\{H,\;0,\;0,\;0\right\} \end{array}$$

with $R_{m}=\frac{\mu_{m}}{\delta f\gamma_{e}\gamma_{p}}\left(\gamma_{e}+\mu_{e}\right)\left(\gamma_{l}+\mu_{l}\right)\left(1+\frac{\mu_{l}}{\gamma_{l}}\right)$.

We identified the infected subsystem, i.e., $\cdot M_{e},\;\cdot M_{i},\;\cdot H_{e}$ and $\cdot H_{i}$. Then, we linearized the infected subsystem and evaluated its Jacobian matrix with the infection-free steady state to identify the transmission (*T*) and transition (*V*) matrix

$$\begin{array}{ccc} F & = & \left(\begin{array}{cccc} 0 & 0 & 0 & \beta_{m}\frac{M_{s}^{*}}{H}\\ 0 & 0 & 0 & 0\\ 0 & \beta_{h}\frac{H_{s}^{*}}{M} & 0 & 0\\ 0 & 0 & 0 & 0 \end{array}\right)\\ V & = & \left(\begin{array}{cccc} -(\theta_{m}+\alpha \;\mu_{m}) & 0 & 0 & 0\\ \theta_{m} & -\alpha \mu_{m} & 0 & 0\\ 0 & 0 & -(\mu_{h}+\theta_{h}) & 0\\ 0 & 0 & \theta_{h} & -(\gamma_{h}+\mu_{h}) \end{array}\right) \end{array}$$

We computed $R_{0}=\rho \left(-FV^{-1}\right)$, where ρ is the spectral radius of the matrix. Note that the parameters presented in Equation (A1) correspond to the ones presented in sensitivity analysis in Figure 5a:

(A1) $$R_{0}=\sqrt{\frac{\beta_{h}\;\beta_{m}\;\theta_{h}\;\theta_{m}}{\alpha \;\mu_{m}\;\left(\gamma_{h}+\mu_{h}\right)\;\left(\mu_{h}+\theta_{h}\right)\;\left(\theta_{m}+\alpha \;\mu_{m}\right)}}$$

Finally, note that if we linearize the infected subsystem and estimate the sign of the eigenvalues as described in Khan and Fatmawati [55], Gbadamosi et al. [56], we can see that the eigenvalues for mosquitoes ($-\left(\theta_{m}+\alpha \;\mu_{m}\right),\;-\alpha \mu_{m}$) and for humans ($-\left(\mu_{h}+\theta_{h}\right),\;-\left(\gamma_{h}+\mu_{h}\right)$) are always less than zero. Even so, for future research, it could be important to identify the effects of the pulse input on the model (1) equilibrium. When adding the pulse input as a sum on the infected systems, the eigenvalues could not always be negative because the $u_{m}\in R$. Thus, there are negative values in which the parameter could generate bifurcations in the system.
